# Draft Genome Sequence of *Bacteroides reticulotermitis* Strain JCM 10512^T^, Isolated from the Gut of a Termite

**DOI:** 10.1128/genomeA.00072-14

**Published:** 2014-02-13

**Authors:** Masahiro Yuki, Kenshiro Oshima, Wataru Suda, Mitsuo Sakamoto, Toshiya Iida, Masahira Hattori, Moriya Ohkuma

**Affiliations:** a Biomass Research Platform Team, RIKEN Biomass Engineering Program Cooperation Division, RIKEN Center for Sustainable Resource Science, Tsukuba, Ibaragi, Japan; b Center for Omics and Bioinformatics, Graduate School of Frontier Sciences, the University of Tokyo, Kashiwa, Chiba, Japan; c Japan Collection of Microorganisms/Microbe Division, RIKEN BioResource Center, Tsukuba, Ibaragi, Japan

## Abstract

Here we report the draft genome sequence of *Bacteroides reticulotermitis* strain JCM 10512^T^, a xylanolytic and cellulolytic bacterium isolated from the gut of a wood-feeding termite. The genome information will facilitate the study of this strain for biomass degradation and adaptation to the gut environment.

## GENOME ANNOUNCEMENT

The gut of wood-feeding termites harbors a complex microbial community, which plays crucial roles in lignocellulose degradation and metabolisms of the host termites (1). Although the majority of the gut bacteria are very difficult to cultivate, molecular studies without cultivation have shown the great diversity of termite gut bacteria, and members of the order *Bacteroidales* are among the major constituents in the gut microbial community (2). Strain Rs-03^T^ (available from the Japan Collection of Microorganisms as JCM 10512^T^) was isolated from the gut of the wood-feeding subterranean termite *Reticulitermes speratus* and designated a novel species, *Bacteroides reticulotermitis* (3). *B. reticulotermitis* strain JCM 10512^T^ is obligately anaerobic, and the cells of this strain are nonpigmented, nonmotile, non-spore forming, Gram-negative staining, and rod shaped. This strain ferments xylan, carboxymethylcellulose, and starch (3).

The genome of *B. reticulotermitis* strain JCM 10512^T^ was sequenced using the Ion Torrent PGM system and Newbler version 2.8 (Roche). A total of 564,185 reads were assembled into 97 contigs with an *N*_50_ length of 137,557 bp and the largest length of 270,264 bp. This assembly resulted in a draft genome sequence of 5,365,278 bp with 22.1× redundancy and a G+C content of 43.3%. Totals of 4,841 protein-coding genes and 64 RNA coding sequences were detected after manual inspection of the annotations using the RAST server (4).

RAST annotation and the following analyses with the CAZy database (5) revealed 108 predicted genes for xylanolytic and cellulolytic enzymes in the genome sequence of *B. reticulotermitis* JCM 10512^T^. There were various genes encoding xylanases homologous to those in the glycoside hydrolase 5 (GH5), -10, -30, -43, -95, and -98 families in the CAZy database; genes encoding the α- and β-xylosidases of GH31, -39, -43, and -97; genes encoding cellulases of GH9; and genes encoding α- and β-glucosidases of GH3 and -97. In addition, the genome had several genes encoding α-1,6-mannanase, β-mannosidase, and arabinofuranosidase. Furthermore, the genome had a starch utilization system locus (*susRABCDEF*) similar to that of *Bacteroides thetaiotaomicron*. The genes *susC*, *susD*, *susE*, and *susF* are involved in starch binding, *susA* and *susB* are involved in starch catabolism, and *susR* is involved in regulation of this gene locus as a transcription factor (6, 7). These results support the hypothesis that *B. reticulotermitis* JCM 10512^T^ utilizes soluble and insoluble polysaccharides, and this ability seems to be advantageous for inhabiting the gut of the termite.

### Nucleotide sequence accession numbers.

The genome sequence of *Bacteroides reticulotermitis* strain JCM 10512^T^ has been deposited in DDBJ/EMBL/Genbank under the accession numbers BAIV01000001 through BAIV01000097.
